# Quality of sleep after COVID-19 infection: a cross-sectional study in the Southern Italy

**DOI:** 10.3389/fpsyt.2024.1428423

**Published:** 2024-09-24

**Authors:** Vincenza Sansone, Silvia Angelillo, Giovanna Paduano, Claudia Pileggi, Carmelo Giuseppe Angelo Nobile, Gabriella Di Giuseppe

**Affiliations:** ^1^ Department of Experimental Medicine, University of Campania, “Luigi Vanvitelli”, Naples, Italy; ^2^ Department of Health Sciences, University of Catanzaro “Magna Gracia”, Catanzaro, Italy; ^3^ Department of Pharmacy, Health and Nutritional Sciences, University of Calabria, Cosenza, Italy

**Keywords:** COVID-19, mental health, sleep quality, social activities, survey

## Abstract

**Introduction:**

This study investigated the quality of sleep in a sample of individuals from Southern Italy after the major waves of the COVID-19 pandemic, with the aim of evaluating how sleep patterns changed.

**Methods:**

A cross-sectional study was conducted between March 2022 and January 2023 and involved adults who had a COVID-19 infection, who were invited to complete a self-administered online questionnaire.

**Results:**

A total of 408 individuals participated in the survey. Overall, 66.4% had a reduction in social relations; 72.1% had an increase in the use of social media; and 86%, 77.2%, and 71.1% reported an extremely severe level of anxiety, stress, and depression, respectively. Almost all of the respondents had a Pittsburgh Sleep Quality Index score (PSQI) ≥5, indicating poor sleep quality. Subjects with a severe or extremely severe depression score, a severe or extremely severe stress score, who had a job, and who had someone close who died because of a COVID-19 infection were more likely to have a high PSQI global score. The use of sleep medication in the past months was significantly higher in those who were older, who had a job, who had a COVID-19 infection in the first and second waves, who had someone close who died from COVID-19, and who did not have changes in social relationships during the pandemic. Moreover, participants with severe or extremely severe depression scores, with severe or extremely severe stress scores, who were women, and who were older had troubles staying awake while engaging in social activities during the past month.

**Conclusion:**

The results bring to light the high prevalence of poor sleep quality among individuals who were infected with SARS-CoV-2. Future research is needed to understand whether these disturbances are still present in the endemic period and whether it is necessary to investigate further determinants that have affected and/or are affecting sleep quality.

## 1 Introduction

The onset of the coronavirus disease 2019 (COVID-19) led to a catastrophic global pandemic (1). Managing the disease became increasingly difficult, prompting measures for containment such as track and trace, self-isolation, quarantine, social distancing, and nationwide lockdowns to curb the spread of infection (2, 3). These measures raised concerns about livelihoods and strained relationships, impacting the overall mental and physical health and well-being of individuals and their families (4). Research studies revealed a link between COVID-19 infection outbreaks and sleep dysfunction (5–8). Jaharmi et al. described sleep changes as a common symptom among COVID-19–infected individuals (9). A meta-analysis reported a global prevalence of about 40% for COVID-19–related sleep disturbances (10), yet it remains a relatively underexplored aspect in COVID-19 research (11). Sleep plays a recognized role in influencing the immune response, and there is evidence indicating that sleep disruptions may lead to immunosuppression (12, 13). Sleep, crucial for optimal organ and mental functioning, became a focal point during the pandemic. Poor sleep, associated with adverse clinical outcomes, posed an additional challenge during this emergency (9).

While the psychological and psychiatric repercussions of pandemic-related measures have been extensively studied, understanding their effects on sleep has been hindered by inconsistent study results (14–16).

Sleep-related manifestations, such as insomnia, can pose persistent challenges. If left untreated, insomnia can become chronic, aggravate pain syndromes and gastrointestinal disorders, and elevate the risk of hypertension, heart disease, and neuroinflammation (17, 18).

Italy has been one of the most affected nations worldwide during the pandemic; however, few studies have been conducted on sleep quality in the Italian population after this particular period. Therefore, we investigated the quality of sleep on a sample of individuals from Southern Italy after the major waves of the COVID-19 pandemic, with the aim of evaluating whether and how sleep patterns changed.

## 2 Materials and methods

### 2.1 Study population, sampling procedure, and data collection

This survey is part of larger research activities that investigated sleep quality among different populations in the Southern Italy (19). A cross-sectional study was conducted between March 2022 and January 2023. All individuals ≥18 years of age, who tested positive for COVID-19, were Italian speakers, and attended several health services (outpatient, clinic, vaccination center, etc.) of the Magna Graecia University of Catanzaro were included in the study. Those younger than 18 years old, those who did not give their consent to participate in the study, and those who had not been infected at least once with SARS-CoV-2 were excluded from the study. Part of the sample had already been involved in a previous study that explored the quality of sleep in a specific population-university students-who were or were not infected with SARS-CoV-2 (19).

The required sample size was calculated before the beginning of the study, considering a 95% confidence interval (CI), an alpha error of 5%, and assuming that 40% of individuals had sleep disturbances (10). Therefore, the minimum sample size was estimated to be 369 participants.

The data were collected by an anonymous, self-administered online questionnaire created with the Google Forms® online application. The questionnaire link, sent via email when the subject arrived at the health services, was accompanied by a brief of the study's purposes so that participants could choose to be involved or not, providing informed consent to take part in the survey. Only those who completed the questionnaire in all its parts were included, as the online form could be transmitted only by completing all answers. Furthermore, the online link could only be sent once to avoid repeated responses from the same participant.

The study protocol was approved by the Ethics Committee of the Magna Graecia University of Catanzaro (Protocol no. 107, 21 April 2022) and was conducted according to the Declaration of Helsinki. Participants did not receive any form of fee or incentive for contributing to this survey.

### 2.2 Survey instrument

The questions were grouped into four sections: (1) sociodemographic characteristics of the participants; (2) information on the contagion of COVID-19; (3) assessment of sleep quality; and (4) assessment of the level of anxiety, stress, and depression.

The variables examined in the sociodemographic section included gender, age, marital status, level of education, occupation, and collection of data on participants regarding the contagion by COVID-19, having been forced or not to mandatory quarantine, having had close individuals who had tested positive or not, having lost someone close due to COVID-19, and effects of the pandemic emergency on their social relationships (decrease/improvement of social contacts and use of social media).

Sleep quality was evaluated by the 19-item Pittsburgh Sleep Quality Index (PSQI) (20, 21). The PSQI is a self-administered questionnaire that appraises the quality of sleep with questions regarding the previous month. The scale is made up of the following seven components: subjective sleep quality, sleep latency, sleep duration, habitual sleep efficiency, sleep disturbances, use of sleep medications, and daytime dysfunctions. By combining the records of each component, we obtained a final score between 0 and 21, where a score ≥5 indicates lower sleep quality, while a score <5 indicates higher sleep quality.

The short version of the Depression Anxiety Stress Scales (DASS-21) was employed to investigate anxiety, stress, and depression (22). DASS-21 is a self-rating scale to describe the frequency and severity of negative effects, in particular, depression is investigated by evaluating dysphoria, anhedonia, lack of incentives, and low self-esteem; anxiety by somatic symptoms of physiological over-arousal and fear response; and stress by irritability, impatience, tension, and arousal levels. Subscale scores are calculated as the sum of the answers to the seven items of all subscales and multiplied by 2 to meet the 42 items of the original instrument. The cutoffs of ≥14 defined moderate levels of depression, ≥10 of moderate anxiety, and ≥19 of moderate stress (23).

### 2.3 Statistical analysis

Results were examined using descriptive (frequencies, means, and standard deviations) and inferential (bivariate and multivariate analyses) statistics with the Stata software version 17 (24).

First, descriptive statistics were performed to summarize the main characteristics of the sample. Second, a univariate analysis was performed using the chi-square test, Student’s t-test, and Fisher’s exact test to evaluate the association between several potential determinants and having poor or good sleep (poor sleep = 0; good sleep = 1). All independent variables considered as potential determinants of the global score (PSQI) (continuous) (Model 1), of having used sleeping medicines in the past month (Model 2), and of having had trouble staying awake while engaging in social activities during the past month the questionnaire (Model 3) were included in the multivariate linear and logistic regression models with the purpose of identifying those predicting these outcomes of interest.

In the multivariate linear model, the following independent variables, which were judged to potentially have influenced the above mentioned outcome (Model 1), were included: gender (male = 0; female = 1); age in years (continuous); marital status (unmarried/widowed/divorced = 0; married/cohabitant = 1); education level (high school = 0; university degree/master = 1); occupation; time of COVID-19 infection (first and second waves = 0; subsequent waves = 1); someone close positive for COVID-19; someone close died from COVID-19; forced quarantine; changes in the social relationship during the pandemic (decreased = 0; stable/improved = 1); change in social media use during the pandemic (stable/decreased = 0; improved = 1); anxiety score (normal/mild/moderate = 0; severe/extremely severe = 1); depression score (normal/mild/moderate = 0; severe/extremely severe = 1); and stress score (normal/mild/moderate = 0; severe/extremely severe = 1). In the multivariate logistic models (Models 2 and 3), the following independent variables were selected for both models: gender (male = 0; female = 1); age in years (continuous); marital status (unmarried/widowed/divorced = 0; married/cohabitant = 1); education level (high school = 0; university degree/master = 1); occupation; time of COVID-19 infection (first and second waves = 0; subsequent waves = 1); someone close who tested positive for COVID-19, someone close who died from COVID-19; forced quarantine; changes in social relationship during the pandemic (decreased = 0; stable/improved = 1); change in social media use during the pandemic (stable/decreased = 0; improved = 1); anxiety score (normal/mild/moderate = 0; severe/extremely severe = 1); depression score (normal/mild/moderate = 0; severe/extremely severe = 1); and stress score (normal/mild/moderate = 0; severe/extremely severe = 1).

Backward stepwise procedures were applied, including in the final models only the characteristics that provided a significant explanation of the outcomes. Adjusted odds ratios (ORs) and 95% CIs were presented in the logistic regression models and standardized regression coefficients (β) and *p*-values in the linear regression model. All statistical tests were two-sided, and a *p*-value equal to or less than 0.05 was considered statistically significant.

## 3 Results

All 408 invited individuals agreed to participate in the survey. The main characteristics of the examined sample are reported in **Table 1**.** **A large majority of participants were women (75.5%), with a mean age of 31.7 (range = 19–64 ± 12.1), and 46.6% were unmarried, widowed, or divorced. Approximately half (46.8%) already had a university degree or master’s, and 41% were employed. Moreover, according to the COVID-19–related data, 61.5% of the participants declared to have had the infection in the third or subsequent waves, while quarantine was imposed on 98.1% of them; in addition, 92.4% declared to have had someone close who tested positive for COVID-19, and 10% had experienced someone close who died from COVID-19 infection. None reported to have been infected more than once. After the third wave, the majority of them (66.4%) had a reduction in social relationships, and 72.1% had an increase in the use of social media. Regarding mental health scores, individuals reported an extremely severe level of anxiety (86%), stress (77.2%), and depression (71.1%). Moreover, when exploring sleep quality, 95.3% of respondents had a PSQI score ≥5 (poor sleep quality). Regarding the single components of the PSQI score, 47.3% of participants reported a very bad subjective sleep quality; approximately half (49.8%) declared a sleep duration of 6 to 7 h per night; more than half (59.1%) reported having used sleep medications in the past month; and almost all subjects in the sample (93.2%) experienced daytime dysfunction in the past month, such as having trouble staying awake while driving, eating meals, or engaging in social activity (**Supplementary Materials**).

**Table 1:** Sociodemographic characteristics, COVID-19, and mental health data of the study population.

Characteristics	Total (*n* = 408)		Poor sleeper (*n* = 389, 95.3%)		Good sleeper (*n* = 19, 4.7%)	
SOCIO-DEMOGRAPHIC	*N*	%	*N*	%	*N*	%
Gender						
Male	100	24.5	93	93	7	7
Female	308	75.5	296	96.1	12	3.9
			*p* = 0.201			
**Age group (years)**	31.7 ± 12.1(range:19–64)^*^		*p =* 0.291			
Marital status						
Unmarried/widowed/divorced	190	46.6	180	94.7	10	5.3
Married/cohabiting	218	53.4	209	95.9	9	4.1
			*p =* 0.587			
Occupation						
Unemployed	241	59.1	228	94.6	13	5.4
Employed	167	40.9	161	96.4	6	3.6
			*p* = 0.396			
Education level						
High school	217	53.2	204	94	13	6
University degree/master’s degree	191	46.8	185	96.9	6	3.1
			*p* = 0.173			
COVID-19 RELATED DATA	*N*	%	*N*	%	*N*	%
Time of COVID-19 infection						
First and second waves	157	38.5	149	94.9	8	5.1
Subsequent waves	251	61.5	240	95.6	11	4.4
			*p* = 0.739			
Someone close positive for COVID-19						
No	31	7.6	28	90.3	3	9.7
Yes	377	92.4	361	95.8	16	4.2
			F-test = 0.17, df = 1			
Someone close died of COVID-19						
No	367	90	348	94.8	19	5.2
Yes	41	10	41	100	0	0.0
			F-test = 0.24, df = 1			
Forced quarantine						
No	8	1.9	6	75	2	25
Yes	400	98.1	383	95.7	17	4.3
			F-test = 0.05, df = 1			
Changes in social relationships during the pandemic						
Improved	7	1.7	7	100	0	0.0
Stable	130	31.9	123	94.6	7	5.4
Decreased	271	66.4	259	95.6	12	4.4
			*p* = 0.768			
Changes in social media use during the pandemic						
Improved	294	72.1	281	95.6	13	4.4
Stable	109	26.7	104	95.4	5	4.6
Decreased	5	1.2	4	80	1	20
			*p =* 0.261			
MENTAL HEALTH	*N*	%	*N*	%	*N*	%
Depression						
Normal	–	–	–	–	–	–
Mild	–	–	–	–	–	–
Moderate	55	13.5	49	89.1	6	10.9
Severe	63	15.4	60	95.2	3	4.8
Extremely severe	290	71.1	280	96.6	10	3.4
			*p* = 0.055			
Anxiety						
Normal	–	–	–	–	–	–
Mild	–	–	–	–	–	–
Moderate	8	2	7	87.5	1	12.5
Severe	49	12	45	91.8	4	8.2
Extremely severe	351	86	337	96	14	4
			*p* = 0.244			
Stress						
Normal	–	–	–	–	–	–
Mild	12	2.9	8	66.7	4	33.3
Moderate	31	7.6	29	93.5	2	6.5
Severe	50	12.3	47	94	3	6
Extremely severe	315	77.2	305	96.8	10	3.2
			*p <* 0.001			

*Mean ± Standard Deviation (Range)

The results of the multivariate linear and logistic regression are shown in **Table 2**. The results of the multivariate linear analysis showed that subjects with severe or extremely severe depression scores, with severe or extremely severe stress scores, who had experienced someone close who died because of COVID-19 infection, and who had a job were more likely to have a high PSQI global score indicating poor sleep (Model 1 in **Table 2**).

**Table 2:** Results of multivariate linear and logistic regression analyses examining the determinants of the outcomes of interest.

°Model 1. Global score of the Pittsburgh Sleep Quality Index (PSQI)				
Variable	Coef	SE	*t*	*p*
*p* < 0.001				
Occupation				
Unemployed	1			
Employed	1.06	0.42	2.51	0.013
Someone close died of COVID-19				
No	1			
Yes	1.55	0.51	3.03	0.003
Depression score				
Normal/mild/moderate	1			
Severe/Extremely severe	1.99	0.54	3.66	< 0.001
Stress score				
Normal/mild/moderate	1			
Severe/Extremely severe	2.49	0.59	4.17	< 0.001
Marital status				
Unmarried/widowed/divorced	1			
Married/cohabiting	0.42	0.41	1.04	0.300
Time of COVID-19 infection				
First and second waves	1			
Subsequent waves	−0.37	0.34	-1.08	0.281
Forced quarantine				
No	1			
Yes	1.76	1.11	1.59	0.112
Someone close positive for COVID-19				
No	1			
Yes	−0.91	0.59	-1.51	0.132
Changes in social relationships during the pandemic				
Decrease	1			
Stable/Improved	−0.54	0.33	−1.66	0.097

^*^Model 2. Those who have taken sleeping medication in the past month				
*p* < 0.001; no. of observations = 408				
Variable	OR	95% CI		*p*
**Age group (years)**	1.06	1.01–1.12		0.031
Occupation				
Unemployed	1			
Employed	11.19	3.89–32.16		<0.001
Time of COVID-19 infection				
First and second waves	1			
Subsequent waves	0.29	1.17–7.51		0.022
Someone close died from COVID-19				
No	1			
Yes	2.96	1.10–7.22		0.031
Changes in social relationships during the pandemic				
Stable/ Improved	1			
Decreased	0.35	0.19–0.64		0.001
Marital status				
Unmarried/widowed/divorced	1			
Married/cohabiting	1.51	0.78–2.90		0.219
Changes in social media use during the pandemic				
Stable/ Decreased	1			
Improved	1.57	0.83–2.95		0.162
Depression score				
Normal/mild/moderate	1			
Severe/Extremely severe	2.47	0.81–7.49		0.111
Stress score				
Normal/mild/moderate	1			
Severe/ Extremely severe	3.25	0.85–12.44		0.085
Anxiety score				
Normal/mild/moderate	1			
Severe/Extremely severe	2.93	0.29–28.88		0.357

^#^Model 3. Those who have had trouble staying awake while engaging in social activities during the past month				
*p* < 0.001; no. of observations = 408				
Variable	OR	95% CI		*p*
Gender				
Male	1			
Female	2.24	1.32–3.81		0.003
**Age group (years)**	1.04	1.00–1.08		0.033
Stress score				
Normal/mild/moderate	1			
Severe/Extremely severe	4.93	1.89–12.80		0.001
Depression score				
Normal/mild/moderate	1			
Severe/Extremely severe	2.59	1.21–5.52		0.014
Marital Status				
Unmarried/widowed/divorced	1			
Married/cohabiting	1.61	0.85–3.04		0.142
Occupation				
Unemployed	1			
Employed	0.57	0.25–1.29		0.181
Time of COVID-19 infection				
First and second waves	1			
Subsequent waves	1.58	0.94–2.65		0.084
Forced quarantine				
No	1			
Yes	2.56	0.53–12.43		0.243
Someone close died from COVID-19				
No	1			
Yes	1.54	0.71–3.35		0.280
Changes in social media use during the pandemic				
Stable/Decreased	1			
Improved	1.28	0.77–2.15		0.344
Changes in social relationships during the pandemic				
Stable/Improved	1			
Decreased	0.64	0.39–1.04		0.074
Anxiety score				
Normal/mild/moderate	1			
Severe/Extremely severe	5.66	0.62–51.44		0.124

°The following variables were deleted by backward elimination procedure: gender, age groups (years), education level, changes in social media use during the pandemic, and anxiety score.

*The following variables were deleted by the backward elimination procedure: gender, education level, forced quarantine, and someone close positive for COVID-19.

^#^The following variables were deleted by the backward elimination procedure: education level and someone close positive for COVID-19.

When exploring behaviors, the use of sleep medication in the past months was significantly higher in those who were older (OR = 1.06; 95% CI 1.01–1.12), who had a job (OR = 11.19; 95% CI 3.89–32.16), who had COVID-19 infection in the first and second waves (OR = 0.29; 95% CI 0.16–0.52), who had someone close who died from COVID-19 (OR = 2.96; 95% CI 1.17–7.51), and who did not have changes in their social relationship during the pandemic (OR = 0.35; 95% CI 0.19–0.64) (Model 2 in **Table 2**). Moreover, the participants with severe or extremely severe depression scores (OR = 2.59; 95% CI 1.21–5.52) or stress scores (OR = 4.93; 95% CI 1.89–12.80), who were women (OR = 2.24; 95% CI 1.32–3.81), and were older (OR = 1.04; 95% CI 1.00–1.08) had significantly higher troubles staying awake while engaging in social activities during the past month (Model 3 in **Table 2**).

## 4 Discussion

Much of the literature emphasizes how behaviors relative to sleep in this historical period have changed and have had an impact, especially on mental disorders (25). The major factors influencing this association are physical illness, separation from loved ones, environmental stresses, social isolation, and pre-existing poor mental health (26–30). This study explored the determinants of sleep quality in individuals who had COVID-19 after major pandemic waves and suggested interesting implications for public health interventions aimed at improving sleep quality.

First, it is very interesting that 95.3% of respondents reported poor sleep quality; these data are higher than those reported in Europe before the COVID-19 pandemic, ranging from 35% to 47% (21, 31, 32). Compared with the quality of sleep in other countries, the results are worse than those reported in Romania (51%), Greece (52.4%), Pakistan (61.5%), and England (68.9%) (33–36).

Therefore, these data are very alarming; indeed, a meta-analysis that investigated the changes in the quality of sleep during the pandemic showed that 57% of the general population suffered a clear worsening in the quality of sleep (37). It is important to underline that disruption of bioelectrical brain activity and neurological symptoms have already been described in patients with COVID-19 infection at the beginning of the pandemic (38). Nevertheless, our results describe a very worrying situation that is in line with the data reported by this research group on university students in Italy during the pandemic, describing a relevant percentage of students (92.8%) as poor sleepers (19). Results suggest that this topic requires the insights that have been given in this paper in order to better understand, in a larger population, including university students who contract COVID-19, what the determinants of poor sleep quality are.

Second, regarding mental health scores, individuals reported extremely severe levels of anxiety (86%), stress (77.2%), and depression (71.1%). These data are more alarming, according to the DASS-21 than those reported in other countries. For example, in Vietnam, the proportion of extremely severe levels of depression, anxiety, and stress was reported to be 3%, 3.6%, and 0.8%, respectively (39), and in the Philippines, 4.2% reported severe to extremely severe depressive symptoms, 11.1% reported severe to extremely severe anxiety symptoms, and 3.9% reported severe to extremely severe stress signals (40). Moreover, results from the general population in Spain also described lower extreme values (2.8% for depression, 4.1% for anxiety, and 3.3% for stress) (41). A significant increase in depression and anxiety has been described from 6 months to 2–3 years after COVID-19 infection in the UK (42). Moreover, a study conducted in six countries described that COVID-19 patients had a higher prevalence of depressive symptoms (43). The lockdown and the prevailing epidemic situation in the Italian region had an impact on people's daily lives and their relationships with others (44), and presumably, this contributed to the variations in mental health problems observed during the COVID-19 pandemic. Indeed, Italy was one of the first and most affected countries worldwide during the pandemic; therefore, uncertainty and social restrictions may have adversely affected mental health. Further investigations are needed to better understand these associations.

The results of the multivariate linear and logistic regression analyses showed that several characteristics were independently associated with the different outcomes of interest. In particular, a higher PSQI global score was associated with depression and stress; indeed, it has been described that the overlapping prevalence rates between mental health symptoms and poor quality of sleep problems point to the likely bidirectional relationships between sleep and psychiatric diseases, particularly when more symptoms are present (such as depression and stress) (9). Moreover, those who had experienced someone close who died from COVID-19 infection were more likely to have a high PSQI global score; in fact, it has been described that poor sleep quality may be associated with trauma or post-traumatic stress disease symptoms (45, 46). Those who had a job were more likely to have a high PSQI global score; these data are inconsistent with other results reporting non-employees to have worse sleep quality during the COVID-19 pandemic (47). However, pre-pandemic results found evidence for reversed relations between work-related stress and sleep quality; in particular, low sleep quality was associated with a worsening in work-related stress over time (48). The use of sleep medication in the past months was significantly higher in those who were older, in those who had a job, in those who had COVID-19 infection in the first and second waves, in those who had someone close who died from COVID-19, and in those who did not change their social relationships during the pandemic. Our results, reporting higher use of sleep medications among the elderly, were confirmed by previous literature describing the elderly as being at higher risk for sleep disturbances (49). The higher risk of sleep problems in those who contracted COVID-19 has been attributed to physical pain and the side effects of medications administered for the treatment of the infection (50). Moreover, participants with severe or extremely severe depression or stress scores, who were women, and who were older, had more troubles staying awake while engaging in social activities during the past month. Evidence of an association between poor sleep quality and poorer physical performance in older adults, in particular women, has been described (51). It has been reported that bedtime and wake time were generally much later in women than in men and that women had significantly shorter sleep duration than men, particularly in individuals aged 30–40 (52).

This study presents some possible limitations that need to be explained when interpreting the results. First, the analysis was based on cross-sectional data, so absolute causal assumptions about the detected relationships between determinants and outcomes are limited. Second, participants may have been influenced to answer in a more “desirable” way, and this may have led to an overestimation of sleep quality and its components. Third, there were potential biases related to the use of self-report questionnaires, such as recall bias, because participants’ responses about sleep quality or mental health status might not be accurate. Fourth, the sample was collected in Southern Italy from a single center and might not be completely representative and may limit generalizability to the Italian population. Fifth, potential confounding factors have not been explored, such as drug use, previous sleep, and mental or psychiatric diseases diagnosed before or after the COVID-19 infection, for which participants were being treated by a psychiatrist or a neurologist. Last, the quality of sleep has also not been assessed with an objective procedure, such as the polysomnographic test; it was only measured with a subjective tool, and this might not indicate the participants’ real quality of sleep.

Despite these limitations, the results have delivered new knowledge on a relevant issue not yet completely explored.

The results of the current study bring to light the high prevalence of poor sleep quality among individuals who were infected by SARS-CoV-2 and add to the recent literature on sleep quality on how the pandemic had an impact on sleep health. Future research is needed to understand whether these disorders are still present in the endemic period and whether it is necessary to investigate further determinants that have affected and/or are affecting sleep quality.

## Data Availability

The raw data supporting the conclusions of this article will be made available by the authors, without undue reservation.
