# Supplementary material for: Quality of sleep after COVID-19 infection: a cross-sectional study in the Southern Italy
Source: Front Psychiatry. 2024 Sep 24;15:1428423. doi: 10.3389/fpsyt.2024.1428423 (PMC11462549; doi:10.3389/fpsyt.2024.1428423)
Supplement: Supplementary file 1 [file Table1.docx]

**Supplementary materials.** PSQI components and factor.

| **COMPONENTS** | **Very good**  **N(%)** | **Fairly good**  **N(%)** | **Fairly bad**  **N(%)** | **Very bad**  **N(%)** |
| --- | --- | --- | --- | --- |
|  |  |  |  |  |
| **1: Subjective sleep quality** |  |  |  |  |
| During the past month, how would you rate your sleep quality overall? | 12(2.9) | - | 203(49.8) | 193(47.3) |
| **2: Sleep latency** | **0** | **1-2** | **3-4** | **5-6** |
| Sum of two response: During the past month, how long (in minutes) has it usually taken you to fall asleep each night and during the past month, how often have you had trouble sleeping because you cannot get to sleep within 30 minutes | 23(5.6) | 126(30.8) | 152(37.3) | 107(26.3) |
| **3: Sleep duration** | **>7 hours** | **6-7 hours** | **5-6hours** | **<5 hours** |
| During the past month, how many hours of actual sleep did you get at night? (This may be different than the number of hours you spent in bed) | 160(39.2) | 203(49.8) | 26(6.3) | 19(4.7) |
| **4: Sleep efficiency** | **>85%** | **75-84%** | **65-74%** | **<65%** |
| (Hours slept/ hours in bed) x 100%  Hour slept: During the past month, how many hours of actual sleep did you get at night?  Hours in bed: sum of two response: during the past month, what time have you usually gone to bed at night? and during the past month, what time have you usually gotten up in the morning? | 193(47.4) | 98(24) | 53(12.9) | 64(15.7) |
| **5: Sleep disturbance** | **0** | **1-9** | **10-18** | **19-27** |
| Sum of 9 response: during the past month, how often have you had trouble sleeping because you wake up in the middle of the night or early morning and have get up to use the bathroom and cannot breathe comfortably and cough or snore loudly and feel too cold and feel too hot and have bad dreams and have pain | 1(0.3) | 138(33.8) | 226(55.4) | 43(10.5) |
| **6: Use of sleep medications** | **Not during past month** | **Less than one a week** | **Once or twice a week** | **Three or more times a week** |
| During the past month, how often have you taken medicine to help you sleep (prescribed or “over the counter”)? | 167(40.9) | 151(37) | 43(10.6) | 47(11.5) |
| **7: Daytime dysfunction** | **0** | **1-2** | **3-4** | **5-6** |
| Sum of two response: During the past month, how often have you had trouble staying awake while driving, eating meals, or engaging in social activity? And during the past month, how much of a problem has it been for you to keep up enough enthusiasm to get things done? | 28(6.8) | 154(37.8) | 135(33.1) | 91(22.3) |
